# Genome streamlining in *Parcubacteria* transitioning from soil to groundwater

**DOI:** 10.1186/s40793-024-00581-6

**Published:** 2024-06-20

**Authors:** Narendrakumar M. Chaudhari, Olga M. Pérez-Carrascal, Will A. Overholt, Kai U. Totsche, Kirsten Küsel

**Affiliations:** 1 Aquatic Geomicrobiology, Institute of Biodiversity, Friedrich Schiller University Jena, Jena, Germany; 2 German Center for Integrative Biodiversity Research (iDiv) Halle-Jena-Leipzig, Friedrich-Schiller-Universität, Leipzig, Germany; 3 Cluster of Excellence Balance of the Microverse, Friedrich Schiller University Jena, Jena, Germany; 4 Hydrogeology, Institute of Geowissenschaften, Friedrich-Schiller-Universität Jena, Burgweg 11, 07749 Jena, Germany

**Keywords:** Candidate phyla radiation, CPR, *Parcubacteria*, Genome streamlining, Metagenomics, Soil-seepage, Environmental change

## Abstract

**Background:**

To better understand the influence of habitat on the genetic content of bacteria, with a focus on members of Candidate Phyla Radiation (CPR) bacteria, we studied the effects of transitioning from soil via seepage waters to groundwater on genomic composition of ultra-small *Parcubacteria*, the dominating CPR class in seepage waters, using genome resolved metagenomics.

**Results:**

Bacterial metagenome-assembled genomes (MAGs), (318 total, 32 of *Parcubacteria*) were generated from seepage waters and compared directly to groundwater counterparts. The estimated average genome sizes of members of major phyla *Proteobacteria*, *Bacteroidota* and *Cand*. Patescibacteria (Candidate Phyla Radiation – CPR bacteria) were significantly higher in soil-seepage water as compared to their groundwater counterparts. Seepage water *Parcubacteria* (*Paceibacteria*) exhibited 1.18-fold greater mean genome size and 2-fold lower mean proportion of pseudogenes than those in groundwater. *Bacteroidota* and *Proteobacteria* also showed a similar trend of reduced genomes in groundwater compared to seepage. While exploring gene loss and adaptive gains in closely related CPR lineages in groundwater, we identified a membrane protein, and a lipoglycopeptide resistance gene unique to a seepage Parcubacterium genome. A nitrite reductase gene was also identified and was unique to the groundwater *Parcubacteria* genomes, likely acquired from other planktonic microbes via horizontal gene transfer.

**Conclusions:**

Overall, our data suggest that bacteria in seepage waters, including ultra-small *Parcubacteria*, have significantly larger genomes and higher metabolic enrichment than their groundwater counterparts, highlighting possible genome streamlining of the latter in response to habitat selection in an oligotrophic environment.

**Supplementary Information:**

The online version contains supplementary material available at 10.1186/s40793-024-00581-6.

## Background

Prokaryotes are susceptible to frequent losses and highly variable fluctuations in genetic content, oftentimes induced by selective environmental pressures [1, 2]. Genome reduction leads to simplified metabolism and lowered energetic requirements for cell duplication [3, 4]. In bacteria, genome sizes are also habitat-dependent [5, 6]. A global survey of genome size distribution suggested that aquatic bacteria harbor smaller genomes than their terrestrial counterparts, as spatially and temporally diverse soil environments likely favor a broader genomic repertoire [7]. Aquatic habitats are often dominated by particularly tiny microbes adapted to oligotrophic conditions, as their high surface-to-volume ratios and superior transport systems render competitive advantages [3]. Such is certainly the case for free-living bacteria of the marine SAR11 lineage [1], some marine *Actinobacteria* [8], and freshwater *Betaproteobacteria* [9].

Many groundwater microbiomes are dominated by taxa belonging to the Candidate Phyla Radiation (CPR), a large evolutionary radiation of bacterial lineages characterized by below-average cell sizes (< 1 μm) and compact genomes (< 1 Megabase pairs - Mbp), except *Gracilibacteria* and *Microgenomates* (> 1.5 Mbp) [10–17]. The genome size range of many CPR classes overlaps with that of obligate symbiont bacteria [13] and membrane-associated intracellular parasites [17], which hints at a symbiotic lifestyle. Microscopic evidence points to an episymbiotic lifestyle for some CPR bacteria that attach to larger bacteria [14, 17, 18], and the very few cultured representatives of this radiation, e.g., *Saccharibacteria* (TM7), have been isolated in association with *Actinobacteria* from humans [19, 20].

Given the lack of CPR isolates, most of what is known about their genome sizes, metabolic potential, and lifestyles derives from environmental metagenomic surveys. In rhizosphere grassland soils, mean genome sizes were 0.61 ± 0.14 Mbp for *Parcubacteria*, 0.57 ± 0.11 Mbp for *Saccharibacteria*, and 0.79 ± 0.12 Mbp for *Doudnabacteria* [21], while in Amazon grassland soils mean genome sizes were 0.5 ± 0.08 Mbp for *Parcubacteria* and 1.1 ± 0.2 Mbp for *Microgenomates* [22]. Unfortunately, this limited data on genome size alone tells us very little about the possible causes of variation in their genome size in different habitats.

Evolutionarily related yet ecologically differentiated microbes can emerge across different habitats as result of genome streamlining [23], as closely related microbes tend to segregate ecologically via accumulation of genetic changes and decreased genetic flow among them [24]. Studies on members of the *Methylophilaceae* family reported increased genome streamlining in cells collected from oligotrophic freshwaters compared to those colonizing sediments [23]. These observations were based on comparative whole-genome sequencing of bacterial isolates derived from habitats lacking representative CPR isolates. Given the rarity of CPR bacteria in soil [15, 25], even deep sequencing may not resolve high-quality genomes making it difficult to directly detect them or their relatives in associated groundwater systems.

Our previous studies have shown that CPR bacteria, especially members of the class *Parcubacteria*, are readily and preferentially mobilized via seepage from forest and pasture soils of the Hainich Critical Zone Exploratory (CZE) in central Germany, and might be further vertically transported through the underlying vadose zone via seepage to reach the groundwater [15, 25, 26]. Given the consistently higher relative abundances of CPR bacteria by one or two orders of magnitude in seepage compared to soils of the groundwater recharge areas [26], we aimed to detect CPR bacterial genomes in seepage. These genomes were used as a proxy for soil CPRs that were related to, or even the source of, CPR taxa found in an earlier published comprehensive groundwater metagenomics analysis of the Hainich CZE [16, 27]. Seepage water was obtained using tension-controlled lysimeters installed in 30 to 60 cm soil depth and from drain collectors installed in the underlying vadose zone, to determine the extent to which near-surface and groundwater microbes [16, 27] were genomically divergent, and whether taxonomic and/or genomic differences were due to gene loss and/or adaptive gain over the course of evolution and in response to selective pressure from the latter habitat.

Our data show that the genomes of many bacterial taxa thriving in groundwater were smaller probably as a consequence of long term evolutionary selection in oligotrophic groundwater. Even the most abundant CPR class (*Parcubacteria*), with extremely small genomes, harbored larger genomes with functional enrichments such as a membrane protein than their groundwater counterparts. Consistent with publicly available *Parcubacteria* genomes from soil and groundwater environments, the results of our study indicate that members of the CPR bacterial class *Parcubacteria* undergo an extensive reduction amid genome diversification possibly upon the transition from near-surface soils to oligotrophic groundwater habitats. However, given the great diversity within the CPR superphylum, further experiments are needed to determine how widespread this phenomenon is in the different subphyla of the CPR superphylum.

## Results

### Elevated abundance of CPR bacteria in soil and vadose zone seepage communities

From a total of 71 seepage water samples obtained from the Hainich CZE analyzed via 16S rRNA amplicon sequencing, we selected twelve samples (six from soil seepage and six from vadose zone seepage) for metagenomic sequencing analysis. These samples represented various distinct clusters visualized through Multidimensional Scaling (MDS) plot (Additional file 1: Fig. S1a, b), based on amplicon sequence variants (ASVs) abundances, and were rich in CPR bacteria to keep minimum sample redundancy and to capture maximum CPR MAGs. In these 12 seepage samples, abundances of *Cand.* Patescibacteria ASVs ranged from 0.7 to 35% and 3.9 to 15% in soil and vadose zone seepage, respectively. *Parcubacteria* (up to 29% soil, 7.9% vadose zone) and *Saccharimonadia* (up to 2.9% soil, 9.3% vadose zone) were the most abundant CPR lineages detected in seepage, followed by *Gracilibacteria* (up to 1.8% soil, 0.3% vadose zone) and candidate division ABY1 (up to 0.8% soil, 0.4% vadose zone; Additional file 1: Fig. S1c).

Due to inaccurate or partial metagenomic detection of CPR bacteria with standard marker gene database-based tools, we decided to detect 16S rRNA small subunit (SSU) reads from metagenomes to get an estimate of CPR abundance. The number of distinct CPR SSU reads resulting from the sequenced metagenomes of these 12 samples exceeded the number of PCR amplicon derived ASVs by a factor of 2.5, with relative abundances ranging from 16.3 to 51.8% and 4.7 to 39.9% in soil and vadose zone seepage samples, respectively (Additional file 1: Fig. S1c, d). Abundances of *Parcubacteria*, *Saccharimonadia*, candidate division ABY1, and *Gracilibacteria* reached up to 40, 3.5, 2.7, and 4.2% in the soil seepage communities, respectively, and 22.9, 11.1, 4.6, and 2.5%, in the vadose zone communities, respectively. *Parcubacteria* was the most abundant CPR bacterial lineage in both habitats.

Seepage water CPR bacterial abundances correlated strongly with previously reported measurements from the same ecosystem [15]. However, contrary to previous reports regarding non-CPR microbial community composition in oligotrophic groundwaters [16], we found *Proteobacteria* to dominate both the soil seepage (21.7–60.8%) and vadose zone seepage communities (32.7–40.8%), followed by *Bacteroidota* (3.2–13% and 6–34.6%, respectively), *Verrucomicrobiota* (3.2–7.4% and 1.2–9.2%, respectively), and *Bdellovibrionota* (0.6–2.3% and 0.9–5.7%, respectively; Additional file 1: Fig. S1c).

### MAGs generated from soil and vadose zone seepage waters

**Fig. 1:**
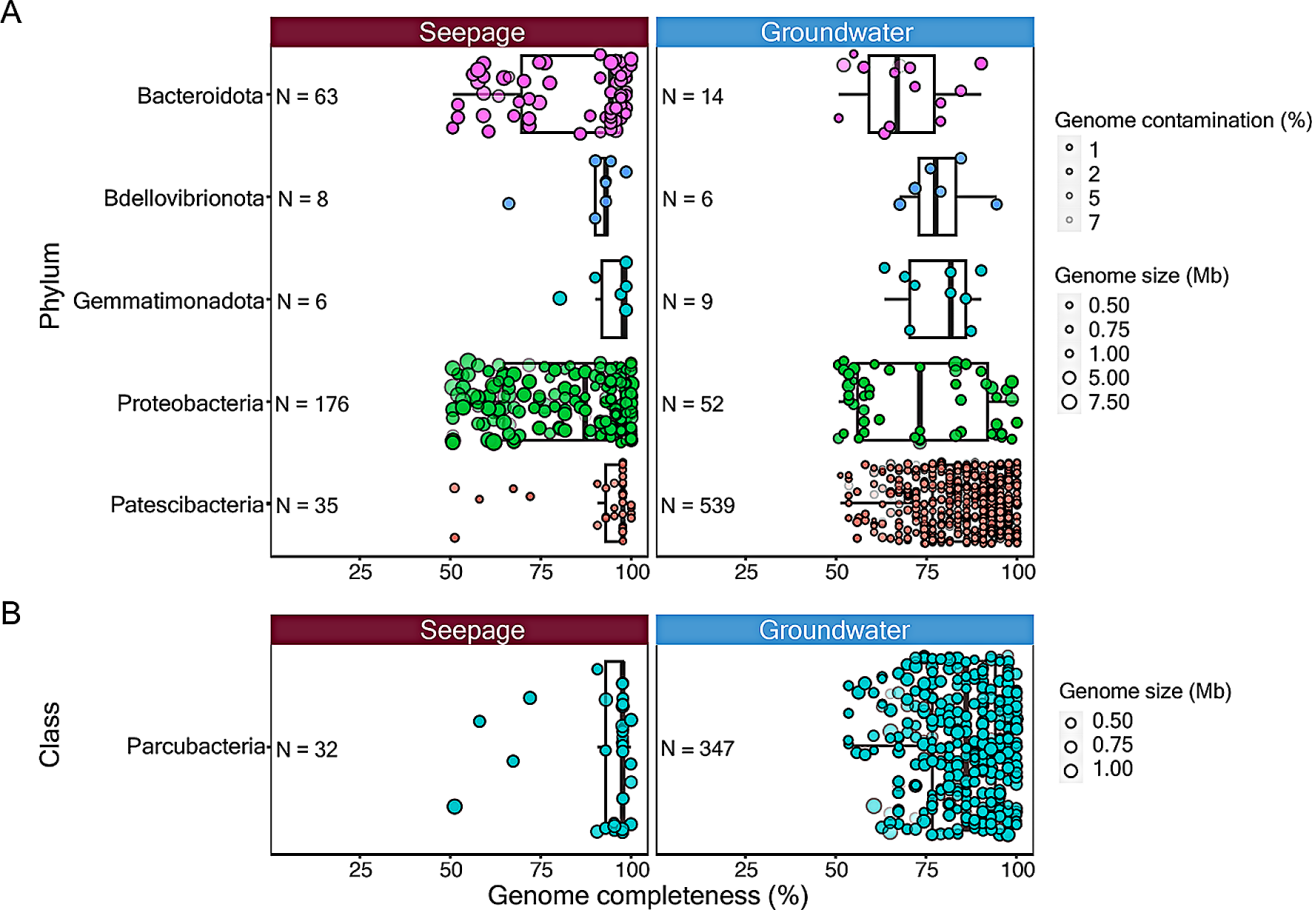
Summary of MAGs recovered from the seepage waters. **A**. Comparison of MAGs generated in this study to previously reported groundwater MAGs [17, 28]. **B**. Comparison of MAGs representing class of *Parcubacteria*. Only phyla and classes represented by a minimum of five MAGs in both data sets were considered, in accordance with MIMAGs standards. Threshold of > 50% genome completeness and < 10% contamination applied to both plots. Circle size indicates genome size while shading intensity indicates extent of contamination

Following appropriate binning and refinement of assembled contigs, 318 non-redundant microbial MAGs were generated from the 12 distinct metagenomic assemblies (Additional file 2: Table S1a, b). Of these, 139 MAGs (5 CPR bacteria) were medium-quality drafts (completeness ≥ 50%, < 90%; contamination < 10%) and 179 (30 CPR bacteria) were high-quality drafts (completeness ≥ 90%; contamination < 5%), as per Minimum Information about a Metagenome-Assembled Genome (MIMAG) standards [28]. With respect to genome completeness and contamination, the quality of seepage-associated MAGs representing bacterial phyla (Fig. 1A), including CPR clades (Fig. 1B), exceeded that of previously published data pertaining to underlying groundwater [16, 27] (Additional file 2: Table S1a). Both medium and high-quality MAGs were generated from both the soil seepage and vadose zone seepage water samples. To get approximate abundances of the species level bins within the binned fraction the metagenomes, the normalized average genome coverages of the seepage MAGs were used (Additional file 2: Table S2).

Of 35 resulting CPR bacterial MAGs, 32 represented *Parcubacteria* (mean estimate genome size 841.4 Kbp ± 156.8) and three *Saccharimonadia* (mean genome size 1328.3 Kbp ± 465.8). We could not generate high-quality assemblies from any other CPR lineages (e.g., *Gracilibacteria*, *Microgenomatia*, candidate division ABY1), likely due to their low abundances or low sequencing depth. Phylogenetic reconstruction based on concatenated alignment of 71 conserved gene-product sequences of all MAGs generated from seepage waters and groundwater showed that CPR (*Parcubacteria*) clades did not segregate based on their source environment. (Additional file 1: Fig. S2, Additional file 3, Additional file 4).

### Larger bacterial genomes in seepage waters than groundwaters

**Fig. 2:**
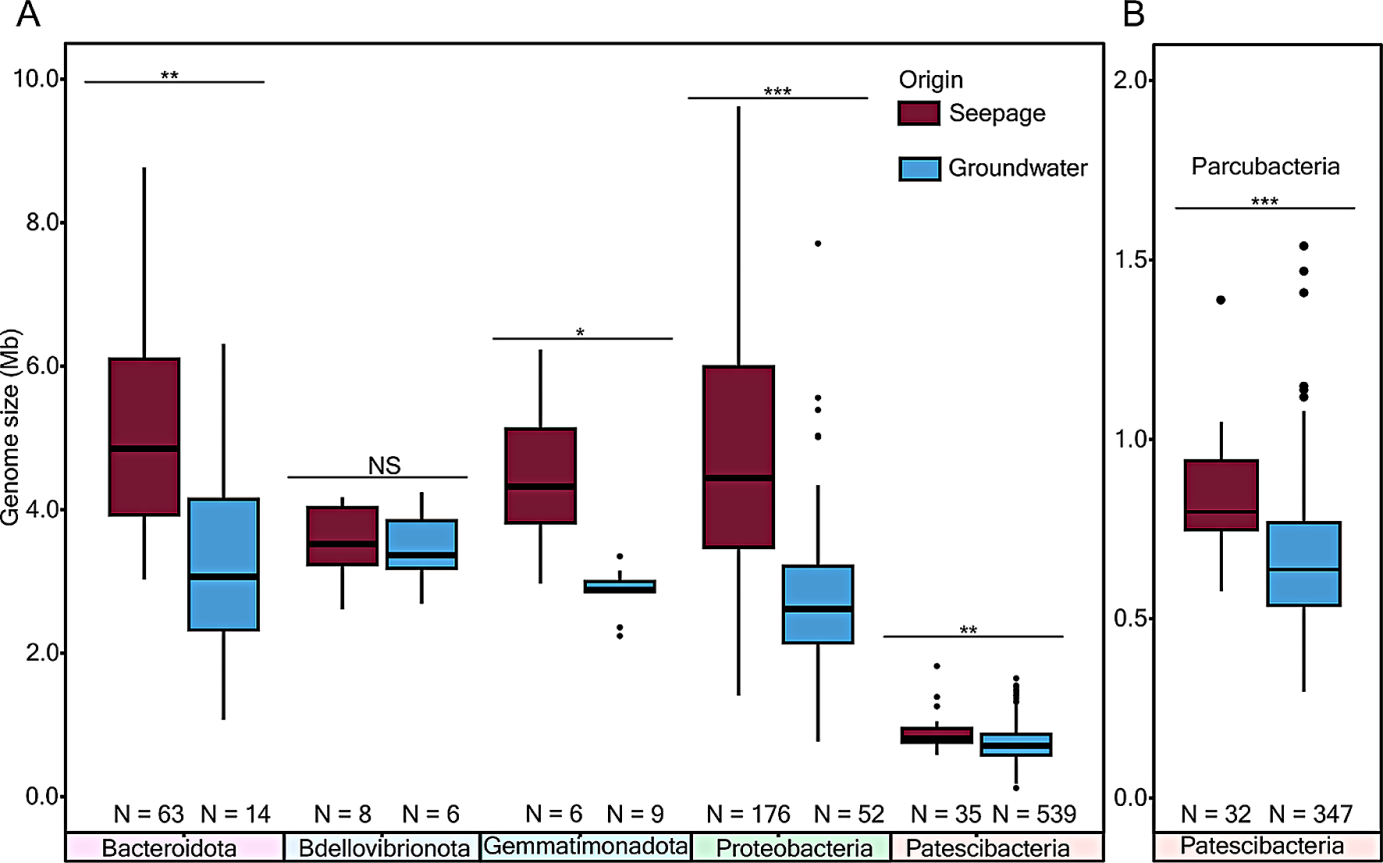
Estimated genome size differences in seepage and groundwater bacteria. **A**. major bacterial phyla, and **B**. *Parcubacteria* class. Statistical significance is denoted as not significant (NS) for *P* > 0.05, * for *P* ≤ 0.05, ** for *P* ≤ 0.01, and *** for *P* ≤ 0.001

Representatives of most major bacterial phyla, including *Bacteroidota*, *Gemmatimonadota*, *Proteobacteria*, and the CPR harbored significantly larger genomes in seepage communities than their counterparts in groundwater communities (Fig. 2A). High-quality Parcubacterial MAGs were significantly larger (*p* = 2.36 × 10^− 5^) in seepage water samples (841.25 ± 157 Kbp) than groundwater samples (661.1 ± 180 Kbp). Mean genomic GC content was comparable (~ 44%) in seepage borne *Parcubacteria* and their groundwater relatives. To test how extended is the streamlining of groundwater CPR genomes compared to seepage CPR genomes, we compared the estimated genomes sizes of other CPR recovered from other studies (29 NCBI datasets with at least five genomes; *n* = 1135, Additional file 2: Table S3). Parcubacterial genomes from seepage have mean genome sizes greater than those from groundwater genomes, with significant differences between seepage and groundwater in eight out of twenty-six groundwater NCBI datasets (Additional file 1: Fig. S3A; Additional file 2: Table S4, S5). Genome sizes of soil CPR were not significantly smaller than seepage genomes.

Next, we tried to identify possible metabolic differences between the general microbial communities of seepage and groundwater. Pathways involved in dissimilatory nitrate reduction to ammonium (DNRA) were restricted to bacterial phyla inhabiting groundwater (Additional file 2: Table S6, S7, and S8) coherent with the dominance of anaerobic ammonium oxidation (anammox) process in groundwaters. Genes involved in the metabolism of polysaccharides like arabinan, cellulose, glucans and xyloglucan were mostly present in seepage bacteria, which agree with a more heterotrophic and carbon-rich environment in seepage. Genes involved in acetoclastic methanogenesis were present in both sites.

### Habitat-distinct traits in closely related *Parcubacteria*

Studying genomically similar microorganisms in different environments using only metagenomics is challenging, especially when the abundance of such targets is particularly low in one or more of the habitats probed. A comparative genome analysis based on mean nucleotide and amino acid identity (AAI, 92%) conducted in parallel with phylogenomic placement (Additional file 1: Fig. S2) and GTDB taxonomy of available MAGs revealed a pair of species that possibly belong to the same genus of *Parcubacteria* – one from seepage and the other from groundwater. This facilitated exploration of genomic and functional differences that may drive the adaptation of microbes transitioning to new habitats.

**Fig. 3:**
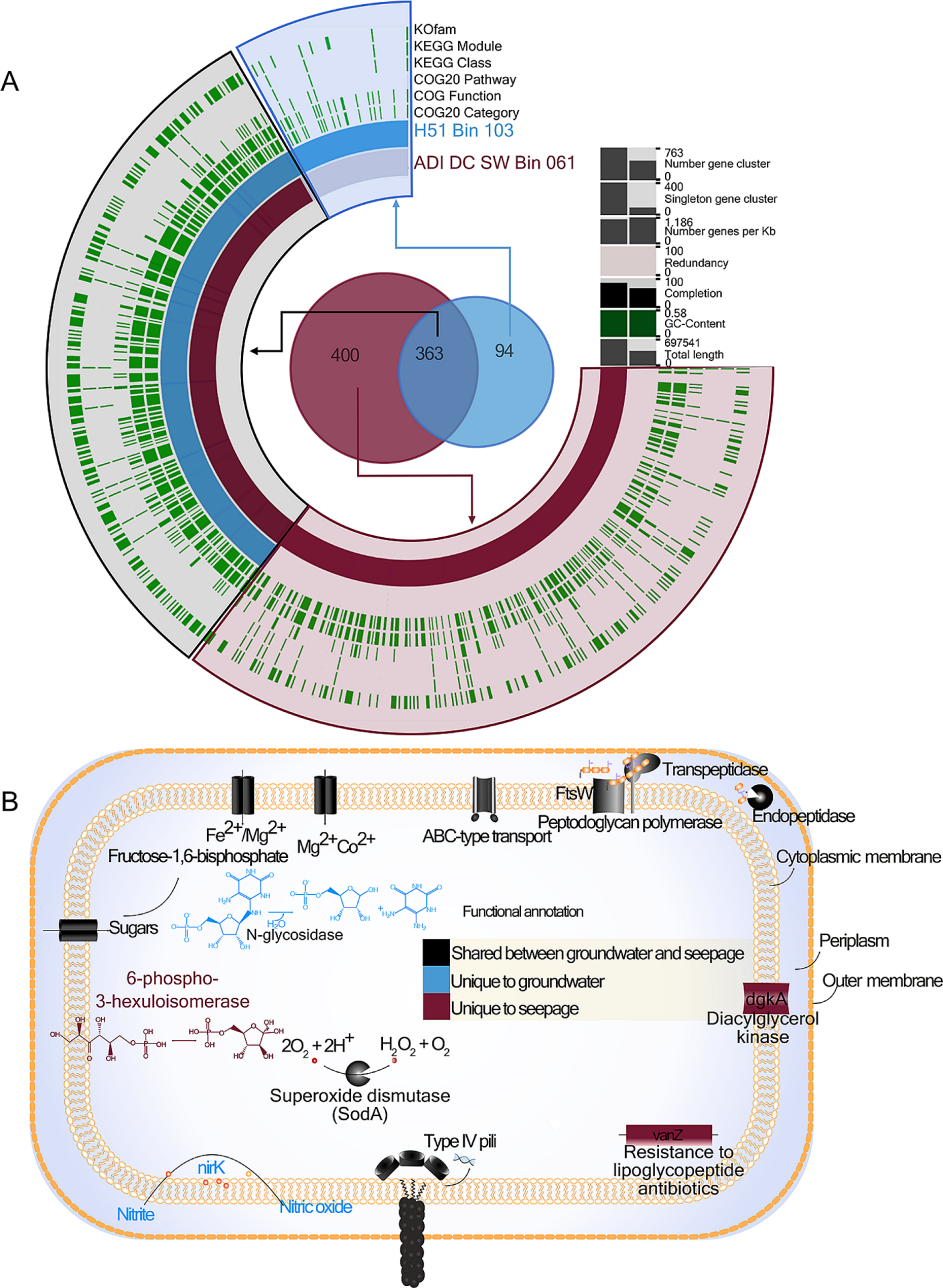
Comparison of shared and genome specific gene functions of representative seepage and groundwater *Parcubacteria*. **(A)** Functional profile of genome-specific and conserved gene clusters between a pair of *Parcubacteria* MAGs. Each bar along the radius of the map represents a distinct gene cluster. Genes from the groundwater MAG (sky blue) and seepage MAG (brown) are shown alongside respective functions (green) derived from COG and KEGG pathways. **(B)** Illustration of genome-specific and shared cellular and metabolic features between the two *Parcubacteria* MAGs

A seepage-borne *Parcubacteria* MAG (ADI-DC-SW-Bin061) belonging to genus C7867-001 was nearly 25% (an estimated genome size of 714 Kbp; 788X depth coverage) larger than that of a closely related groundwater-borne *Parcubacteria* MAG (H51-Bin103; an estimated genome size of 539 Kbp; 32X depth coverage) of the same genus. However, estimated completion levels were only 97% and 72%, respectively (Table 1, Additional file 2: Table S2). Mean AAI between these MAGs was 92.44%. Nearly half of all genes identified in these Parcubacterial MAGs were unannotated against KEGG or COG, most of which were deemed hypothetical proteins. There were 400 genes unique to the seepage *Parcubacterium* genome and 94 gene clusters unique to its groundwater counterpart (Fig. 3A). The 363 genes shared by the two genomes accounted for 79% of the groundwater-borne MAG’s gene content.

**Table 1:** Comparison of features of seepage (this study) vs. groundwater [17, 28] genome of Parcubacterial genus C7867-001

Source	Seepage	Groundwater
Genome name	ADI-DC-SW-Bin061	H51-Bin103
Assembly size (Kbp)	697.6	388.38
Estimated genome size (Kbp)	713.96	538.7
No. of contigs	2	50
Coding genes	775	438
Pseudogenes	38	41
Percentage of pseudogenes	4.9%	9.4%
tRNAs	43	25
GC content	57.9	58.2
Genome completeness	97%	72%
Genome contamination	0%	0%
Fraction of read coverage within binned genomes in the respective sample	35% in vadose zone water sample DC_SW_02_SPN_11032020	0.5% in groundwater well H51

A detailed screening of gene functions revealed the presence of both common and specific habitat-derived structural and metabolic features. A sugar transporter, ion transporters for iron and magnesium, an ABC-type transport system, and superoxide dismutase were encoded by both genomes. The seepage Parcubacterium encoded unique proteins for phospholipid and sugar metabolism as well as an antibiotic resistance protein. Although this MAG encoded additional unique genes compared to its closest relative in groundwater, these genes were also present in other groundwater MAGs of the same genus (Additional file 2: Table S9). Sugar utilization functions were more abundant in seepage-borne CPR bacterium, linking to a more heterotrophic, nutrient rich environment wherein opportunities to utilize sugar intermediaries as energy sources abound. Incomplete pathways for the metabolism of methane, vitamins, cofactors, and amino acids were also detected in the seepage-borne CPR bacterial genome.

In our extended pathway analysis of 12 *Parcubacteria* MAGs surrounding the two compared *Parcubacteria* in the phylogenetic tree, we observed that the groundwater MAG and its phylogenetic neighbors derived from groundwater encoded an enzyme that reduces nitrite to nitric oxide (*nirK*, K00368). While this gene is common to groundwater CPR bacteria [14, 16], it was not detected in seepage CPR bacteria (Fig. 3B, Additional file 2: Table S9). This is a unique example of functional and genomic divergence between two closely related CPR bacteria inhabiting different, yet interconnected, environments. In addition, a gene encoding N-glycosidase was unique to the groundwater-borne CPR bacterial genome. This enzyme plays a role in the cleavage of N-glycosidic bonds of riboflavin intermediates [29]. While pathways involved in the biosynthesis of riboflavin have been reported in groundwater CPR bacteria [17], aspects pertaining to its catabolism (e.g., use of intermediates as an energy source in low nutrient environments) remain poorly understood.

### Genome streamlining in groundwater *Parcubacteria*

**Fig. 4:**
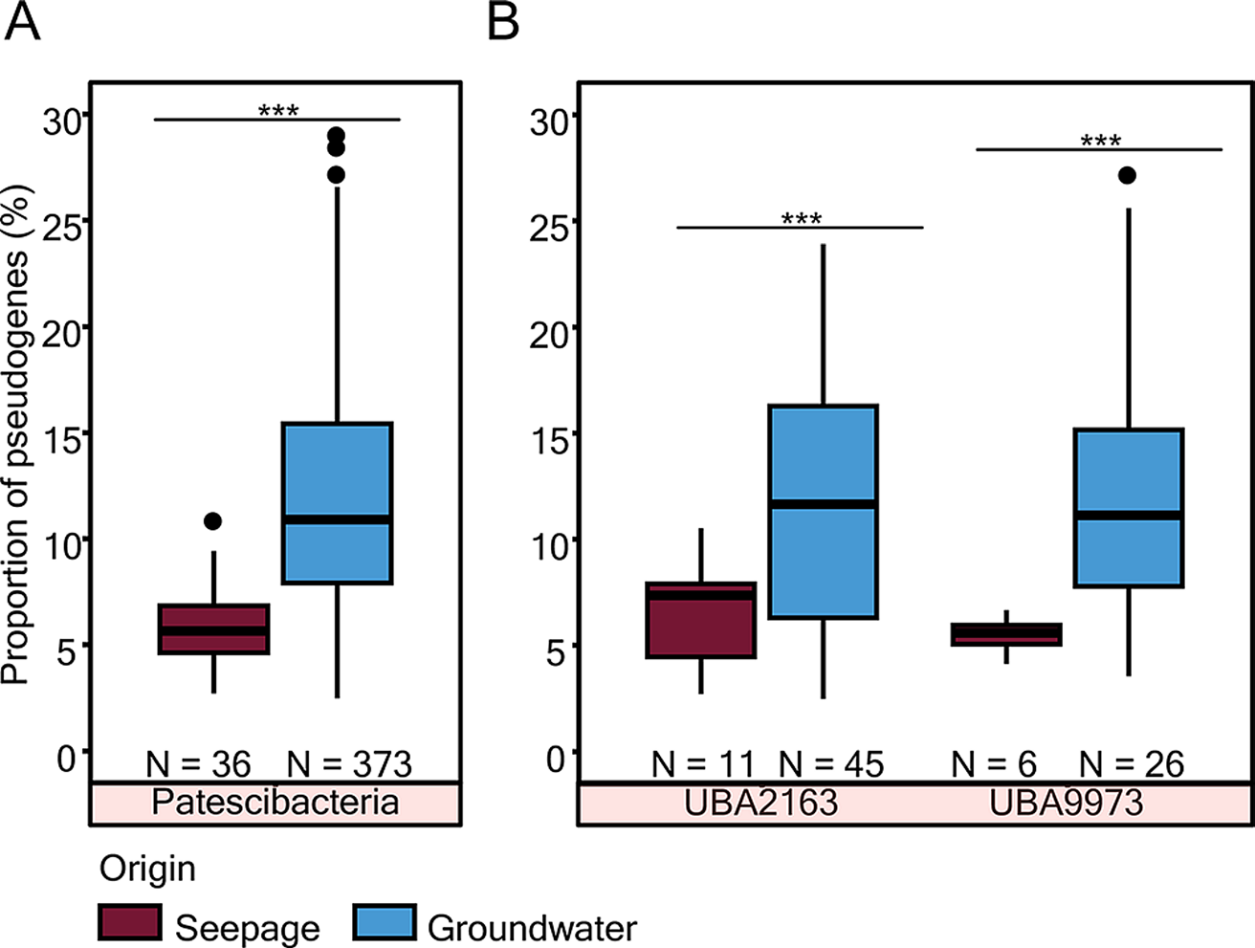
Comparison of pseudogene frequencies in MAGs derived from seepage and groundwater. The percent contribution of pseudogenes is compared for MAGs of CPR bacteria (**A**), and families of *Parcubacteria* (**B**) found both in seepage (this study) and groundwater [17, 28]. Statistical significance is denoted by * for *P* ≤ 0.05, ** for *P* ≤ 0.01, and *** for *P* ≤ 0.001

To determine whether differences in genome size resulted from habitat-dependent streamlining, we quantified pseudogenes i.e. genes in the process of being lost or becoming non-functional. As was expected, the seepage-borne MAG harbored a smaller fraction of pseudogenes (4.9% of coding genes) than the groundwater MAG (9.4% of coding genes; Table 1). A general comparison of all CPR bacterial MAGs showed a similar trend, with groundwater-borne MAGs bearing significantly more pseudogenes than their seepage counterparts (Fig. 4A). This disparity is likely a consequence of genome reduction and loss of metabolic function in groundwater CPR bacteria, which might have rendered improved fitness to the oligotrophic conditions and symbiotic lifestyle. Within groundwater CPRs, the larger genomes showed more number of pseudogenes (p = n.s.) but a lower proportion of pseudogenes (*p* = 4.2 × 10 − 5) to total genes in these genomes (Additional file 1: Fig. S4). suggesting that existing coding genes are replaced with pseudogenes during evolution. It is well known for other bacteria that during the first stages of parasitism, microbes are characterized by an increased proportion of pseudogenes, while this is not the case for free-living and streamlined oligotrophs [3]. Therefore, we tried to investigate such lifestyle in seepage derived CPR bacteria by the co-occurrence patterns with other bacteria based on their metagenomic MAG abundances. We found, in particular, *Parcubacteria* species formed specific pairs with only five non-CPR species from phyla, *Chloroflexota* (1), *Chlamydiota* (1), *Gemmatimonadota* (1) and *Proteobacteria* (2) that may serve as potential hosts (Additional file 1: Fig. S5, Additional file 2: Table S9) while majority of seepage CPRs lacked such co-occurrences.

Low GC content is typically an indicator of limited nitrogen availability and genome streamlining [3, 30, 31], Although, we did not observe significant differences in the GC content of the seepage vs. groundwater CPR MAGs in general (Additional file 1: Fig. S6), MAGs from one particular genus of *Parcubacteria* (C7867-001) derived from groundwater exhibited 8.9% lower (*p* = 0.023) mean genomic GC content than their seepage counterparts).

## Methods and materials

### Sample collection, DNA extraction, and metagenomic sequencing

Seepage samples were obtained from forest and pasture areas in the Hainich CZE (Collaborative Research Center AquaDiva [32]. Those locations are part of a topographic groundwater recharge area (eastern Hainich low-mountain slope) with mixed beech forest at the summit and shoulder position, and pasture in the upper midslope position. The dominant soil types at our sampling sites are cambisols and luvisols, predominantly developed from marine limestone-mudstone alternations of the Middle Germanic Triassic [28, 33].

Six tension-controlled lysimeters installed at the topsoil/subsoil and subsoil/parent rock interfaces at roughly 30 cm to 60 cm below the soil surface were used to collect soil seepage samples as described in Lehmann et al., 2021 [33, 34]. In addition, six collectors were used to sample the free drainage percolating through the vadose zone at depths ranging from 97 to 169 cm. All 71 samples were collected between December 2019 and March 2020. Filtration of soil seepage water and vadose zone seepage water samples was accomplished using 0.1 μm membrane filters (Supor®, Pall). Filters were immediately stored at -80 °C. DNA extraction was carried out from the filters using a DNeasy® PowerSoil® kit in accordance with manufacturer’s protocols (Qiagen, USA). Shotgun metagenomic sequencing was carried out on selected 12 samples (Additional file 1: Fig. S1) using an Illumina NovaSeq 6000 SP Reagent kit (v1.5; 300 cycles) on an Illumina NovaSeq6000 sequencing platform. Metagenomic reads, assemblies and MAGs were deposited in the NCBI BioProject PRJNA1025359. The methodology for 16S rRNA amplicon sequencing was similar to previous publications from this sampling site [30].

### Read quality filtering, metagenomic assembly, genome binning, and bin refinement

Metagenomic sequencing yielded an average of 78.8 ± 7.7 million reads per sample. Following quality filtering via the bbduk script (BBMap version 38.96) [35], only high-quality reads were retained. Read error corrections were processed using bbnorm (BBMap) [35] followed by *de novo* assembly using SPAdes (v3.13.0) (using --meta mode) [36]. Contigs longer than 1,000 bp were binned using maxbin2 [37], metabat2 [38], and binsanity [39]. Metawrap [40] refinement (using filters -c 50 -x 10) was then carried out to refine bins obtained from the three binning algorithms and obtain the best representative MAGs. Bins (MAGs) were manually refined via visual inspection of contig coverage and sequence composition profiles, using the Anvi’o (v.7) suite [41] to further improve the quality of refined genomes. A final genome-quality assessment was carried out using the CheckM [42] workflow (v.1.1.3) with a lineage-specific set of marker genes for all CPR bacteria. Only MAGs having at least 50% genome completeness and at most 10% redundancy/contamination were retained for subsequent comparisons. In addition, the estimated genome size for each MAG was calculated based on its assembly length by taking into account its completeness, and redundancy.

### Taxonomic annotation and phylogenetic analysis of MAGs

Taxonomic annotations of the MAGs selected for analysis were carried out with GTDB-Tk [43] (v1.5.1) using GTDB (release 202) as a reference database [43]. A Maximum-likelihood (ML) phylogenetic tree with seepage (*n* = 318) and groundwater (*n* = 862, non-CPR and CPR bacteria) MAGs was constructed based on concatenated alignments of the amino-acid sequences of 71 single-copy core genes and using iqtree2 (v.2.0.3) and the WAG substitution model (1,000 bootstrap replicates) [44, 45]. The single-copy genes were identified and concatenated using the *anvi-get-sequences-for-hmm-hits* command from Anvi’o (v.7). Gaps in the alignment present in more than 50% of the MAGs were removed using trimAL (v1.4.rev15) [46].

### Comparative genomics of CPR bacterial MAGs from near-surface and groundwater communities

The average amino-acid identities (AAI) of all possible pairs of CPR bacterial MAGs from seepage (*n* = 35) and groundwater (*n* = 584) were calculated using EzAAI (v.1.2.0) [47]. Groundwater MAGs were retrieved from two previous studies (Open Science Framework (OSF) repositories: https://osf.io/wq7tr/, https://osf.io/rp5j3/, and https://osf.io/4ceqs/) [16, 27]. A pair of CPR bacteria MAGs yielding an AAI value of 92.44% (the highest value observed among all comparisons) was selected for in-depth comparative analysis of genetic content and metabolic potential. One of these MAGs arose from a seepage sample, the other from a groundwater sample. Contigs from these Parcubacterial MAGs were processed with Prodigal [48] (v.2.6.3) to identify open reading frames. All protein-coding genes from the two genomes were clustered using the Anvi’o pan-genome suite with default parameters. COG [49] and KEGG [50] functions were annotated using respective databases within Anvi’o. Reverse translated DNA amino acid sequences of the two Parcubacterial MAGs were mapped to the KEGG database using the bidirectional best blast-hits method within the KAAS web server [51], and output was manually screened for individual genes specific to respective genomes. An additional functional gene screening was performed using DRAM (v.1.4.6) [52]. We used DRAM to assess the functional differences between the common phyla found in seepage and groundwater (i.e., *Bacteroidota*, *Bdellovibrionota*, *Gemmatimonadota*, *Proteobacteria*, *Verrucomicrobiota* and *Cand*. Patescibacteria). Pseudogenes were identified using Pseudofinder [53] (v.1.1.0), and counts were normalized to percentage of total annotated genes to simplify direct comparison.

### Co-occurrence network among seepage MAGs

We utilized normalized average genome coverages of MAGs generated in this study to construct a co-occurrence network with proportionality cut-off (Rho) of 0.9 to filter highly significant network connections using the python library networkx (v.3.1, https://networkx.org/ ). We visualized this network using Gephi (v.0.10) [54].

## Discussion

Our experimental approach afforded the ability to study microbes transitioning from soil to underlying groundwater habitats, a feat hitherto achieved only in pelagic waters and sediments [1, 23, 55]. By focusing on differing genomic characteristics between seepage and groundwater-borne microbes, we confirm the occurrence of closely related microbes bearing unique genetic content that supports contrasting lifestyle strategies in these interconnected yet distinct habitats.

Seepage water connects surface habitats like soil to the underlying unsaturated vadose zone and the vadose zone to the groundwater. Samples collected from both soil and vadose zone seepage water were dominated by CPR bacteria, with relative abundances reaching 50.4% and 41.1%, respectively, and *Parcubacteria* accounting for up to 40 and 22.9% of the microbial population, respectively. Remarkably, CPR bacteria accounted for a mere 0.55% of forest soil microbial relative abundance. Similarly, CPR bacteria belong to the rare biosphere of rhizosphere-associated grassland soils [21].*Parcubacteria* were up to three orders of magnitude more abundant in soil seepage, whereas members of *Actinobacteria* and *Acidobacteria*, which tend to adhere to soil matrices, were underrepresented in seepage waters [15]. Ergo, the transition of microbial communities from soil to groundwater appears to favor particular taxa, possibly due to the cellular attachment to soil matrices, surface charges, and/or other hitherto unresolved specific traits or lifestyle determinants [26].

We exploited this disparity in mobility behavior and generated 318 distinct bacterial MAGs from seepage waters. The availability of several seepage water associated MAGs, presumably originating from overlying soil, and 964 groundwater MAGs from two previous studies (Additional file 2: Table S1a) [16, 27] facilitated highly informative comparative analyses of bacterial genomes originating from different habitats. Within the same lineage, seepage bacteria tended to maintain larger genomes than groundwater denizens (Fig. 2A). This trend was prominent in *Proteobacteria* and the CPR class *Parcubacteria* (Fig. 2B). Inter-habitat (seepage vs. groundwater) differences in CPR bacterial genomes were more pronounced than intra-habitat differences (various locations within a seepage area or about a groundwater transect). Overall, our results suggest that the transition of CPR bacteria from complex, heterogeneous surface soil environments to more consistent and oligotrophic groundwaters is accompanied by a reduction in genome size, for these likely episymbiotic bacteria [14]. This trend was confirmed by our comparative analysis of genome sizes of CPR class *Parcubacteria* MAGs derived from published surface and groundwater environmental metagenomes, whereas MAGs obtained from soil and seepage environments showed similar genome sizes (Additional file 2: Table S3-S5) suggesting a tight link between microbial communities of these samples.

Evidence in favor of the theory of genome streamlining in evolutionary-related microbes is beginning to accumulate. However, little is known about this process in groundwater [3, 23]. Between two CPR (*Parcubacteria*) bacterial MAGs of the same genus (C7867-001, AAI = 92.44%), one retrieved from seepage and the other from groundwater, a greater number of genes were exclusive to the seepage-borne MAG than its groundwater counterpart. While our interpretations are somewhat limited by incomplete assembly, this could result from the shedding of unnecessary genes upon transitioning to oligotrophic groundwaters scarce in energy sources. The annotated genes, unique to the seepage *Parcubacteria* C7867-001 and genetically related MAGs isolated from the same source, encode a diacylglycerol kinase, a membrane protein known to play a key role in phospholipid metabolism and bacterial survival under variable osmotic conditions [56], and an antibiotic resistance protein that prevents the binding of lipoglycopeptide antibiotics [57]. The nitrite reductase gene (*nirK*, K00368) was unique to groundwater Parcubacterium and its neighboring *Parcubacteria* from groundwater in the phylogenetic tree (Additional file 2: Table S9) likely in response to local exposures to nitrate and/or nitrite. The products of this gene might also potentially contribute to denitrification processes in groundwater [12] but in the absence of the NO reductase gene, this gene might only be limited to generating NO as a toxic agent or as a signaling molecule. Type-IV pili, typically responsible for natural competence and extracellular DNA uptake [58], were present in both *Parcubacteria*. 

Genome streamlining is an adaptive strategy used by bacteria to save energy. Cells encountering deleterious and/or energy limited conditions shed genetic content and machineries whose upkeep is no longer energetically worthwhile. We observed greater fractions of suspected pseudogenes in the groundwater MAGs. Pseudogenes accounted for roughly 10 and 5% of the coding genes in the genome of the groundwater and seepage borne *Parcubacteria*, respectively. Most often, pseudogenes were functional in the past but underwent mutational changes resulting in their removal over the course of evolution [59]. The greater fraction of such genes in groundwater microbes, is indicative of a higher probability of gene loss and further genome streamlining possibly due to exposure to other host organisms in oligotrophic groundwater.

Bacterial genome size is oftentimes correlated with genomic GC content, with compact genomes of obligate endosymbionts presenting the lowest GC contents [60]. Groundwater borne CPR bacterial MAGs of genus C7867-001 exhibited 8.9% lower (*p* = 0.023) mean genomic GC contents than their seepage relatives. Given the higher production cost of guanine and cytosine and greater intracellular availability of adenine, tyrosine, and uridine [61, 62], low GC content is both energetically favorable and a bolster to fitness in oligotrophic environments.

Co-occurrence patterns hinted at a numerous potential hosts in groundwater, such as members of phyla *Nanoarchaeota*, *Bacteroidota*, MBNT15, *Bdellovibrionota*, *Nitrospirota*, and *Omnitrophota* [16]. Thus, CPR bacteria might form transient attachments to hosts in groundwater for limited periods of time [16]. This does not seem to be true in the co-occurrence patterns in seepage as very few (5) direct isolated pairing between CPR and non-CPR MAGs was observed. However, we cannot rule out the possibility that this lack of co-occurrences is due to fewer available MAGs, fewer samples, or low sequencing depth of the less abundant bacteria and thus lower chances of finding random patterns.

Ultimately, the results of this investigation demonstrate that CPR bacteria, characterized by ultra-compact genomes and minimal biosynthetic and metabolic potential, undergo environmental selection (in this case oligotrophic groundwater ecosystem) at an evolutionary timescale. The observed difference of 11% less genes and greater proportion of pseudogenes in groundwater borne CPR bacteria than their seepage counterparts both demonstrate genome streamlining favored by an oligotrophic environment. The exclusive presence or absence of specific genes in *Parcubacteria* populations from seepage and groundwater exemplifies niche selection by the respective environments over the evolutionary timescales.

### Electronic supplementary material

Below is the link to the electronic supplementary material.

Supplementary Material 1

Supplementary Material 2

Supplementary Material 3

Supplementary Material 4

## Data Availability

All the seepage metagenomic reads and corresponding assemblies are available under NCBI BioProject PRJNA1025359 (https://www.ncbi.nlm.nih.gov/bioproject/1025359). All the seepage MAGs are available from The Open Science Framework (OSF) public repository. The subset of groundwater MAGs used for the study are available from previous publications [16, 27] and the rest from the OSF public repositories https://osf.io/wq7tr/, https://osf.io/rp5j3/, and https://osf.io/4ceqs/. The bioinformatics pipeline scripts used for processing the metagenomes are available at the GitHub repository: https://github.com/waoverholt/nextflow_metagenomic_binning.

## Declarations

### Additional information

Correspondence and requests for materials should be addressed to Kirsten Küsel.
