# Supplementary material for: Genome streamlining in Parcubacteria transitioning from soil to groundwater
Source: Environ Microbiome. 2024 Jun 20;19:41. doi: 10.1186/s40793-024-00581-6 (PMC11188291; doi:10.1186/s40793-024-00581-6)
Supplement: Supplementary file 1 — Supplementary Material 1 [file 40793_2024_581_MOESM1_ESM.pdf]

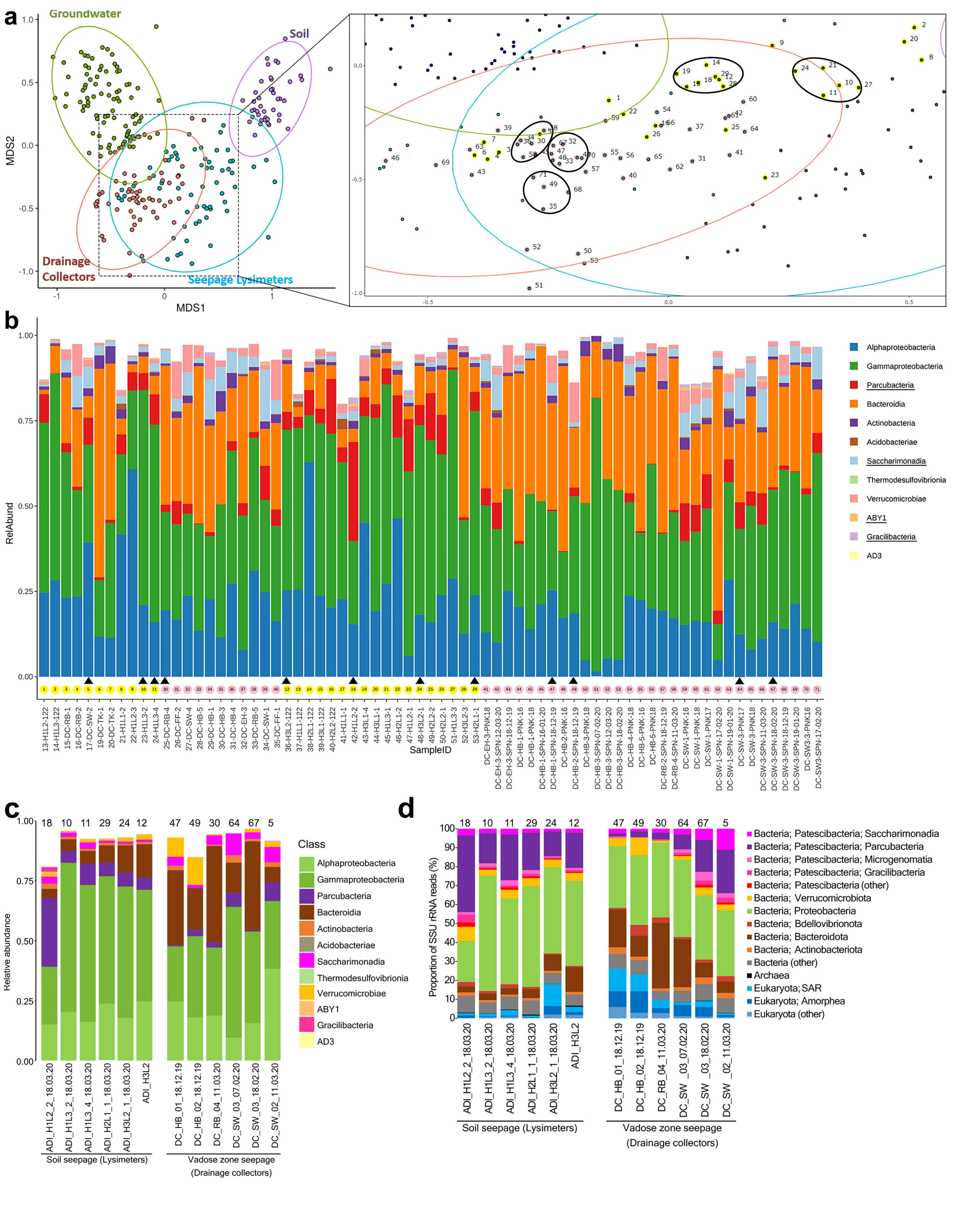

Figure S1: Microbial community composition of seepage waters. Multidimensional Scaling (MDS) clustering of microbial community profiles of various environmental samples to identify closely related seepage samples and select the representatives for metagenomics sequencing (**a**). The selected samples among the seepage samples (filled triangles) with their relative abundances (**b**). Relative abundance of Amplicon Sequence Variants (ASVs) obtained from 16S rRNA amplicon sequencing (**c**) and the fraction of metagenomic reads mapped to the SSU rRNA gene (d). Sample numbers shown above the bars in barplots (**c**) and (**d**) correspond to the selected samples in (**a**) and (**b**).

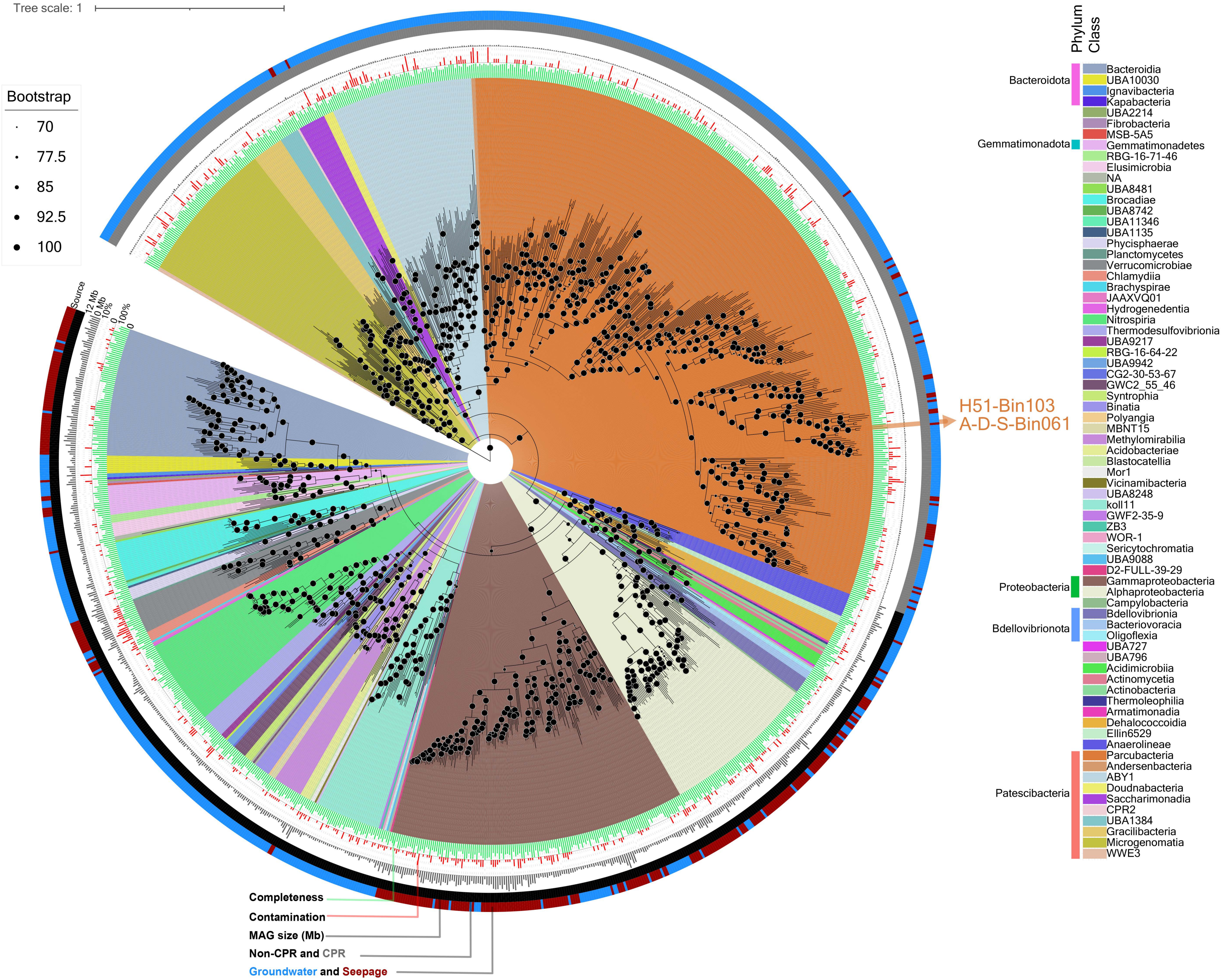

Figure S2: Phylogenetic placement of MAGs generated in this study and the groundwater MAGs from the previous studies [17,28] based on concatenated protein sequence alignments of a set of bacterial single-copy core genes (see Methods for more details). A groundwater Parcubacteria MAG (H51-Bin103) and, a MAG phylogenetically neighboring to the seepage borne Parcubacteria MAG (ADI-DC-SW-Bin061) are labeled.

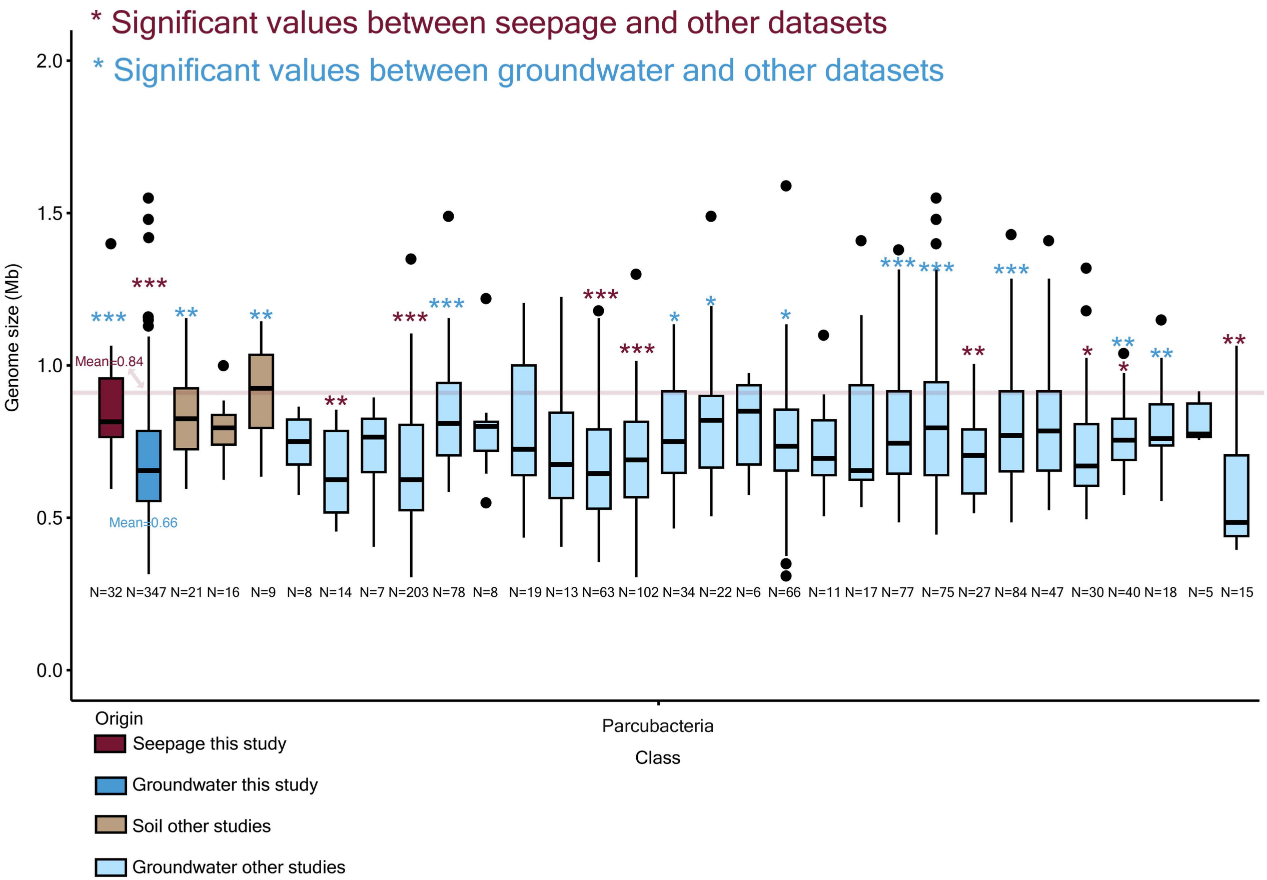

Figure S3: Estimated genome size differences in Parcubacteria MAGs derived from seepage, groundwater, and other related public microbiomes. Statistical significance is denoted as * for P ≤ 0.05, ** for P ≤ 0.01, and *** for P ≤ 0.001.

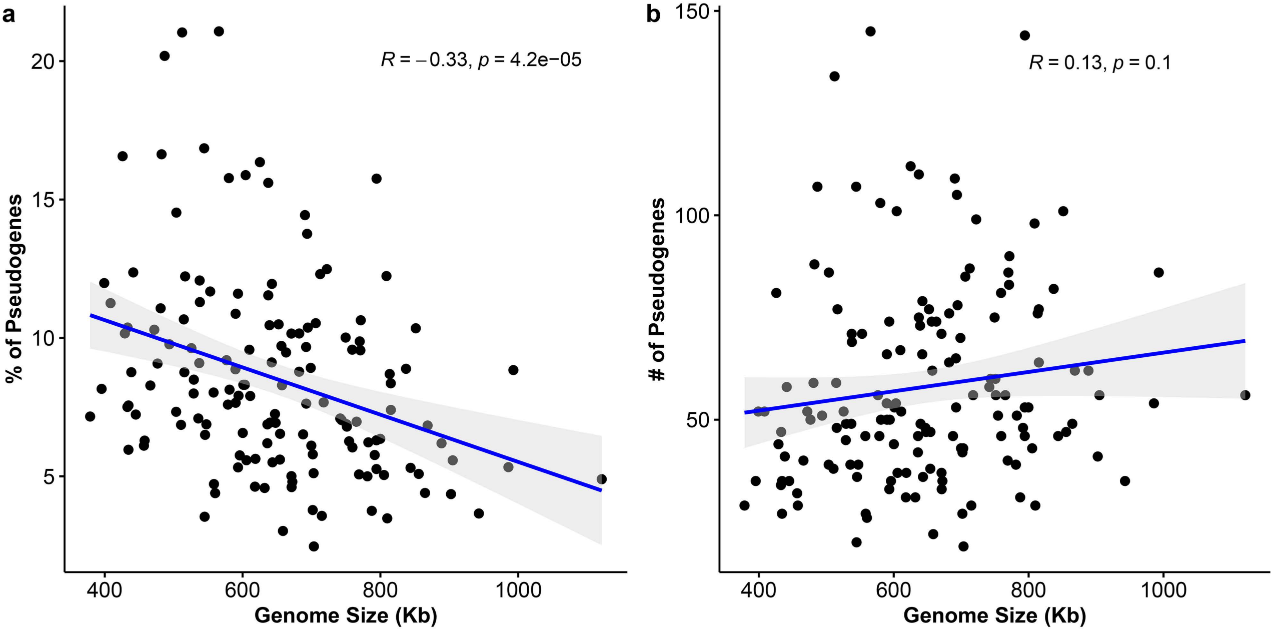

Figure S4: Distribution of the proportion of pseudogenes (**a**), and the number of pseudogenes (**b**) against genome sizes of groundwater CPR MAGs.

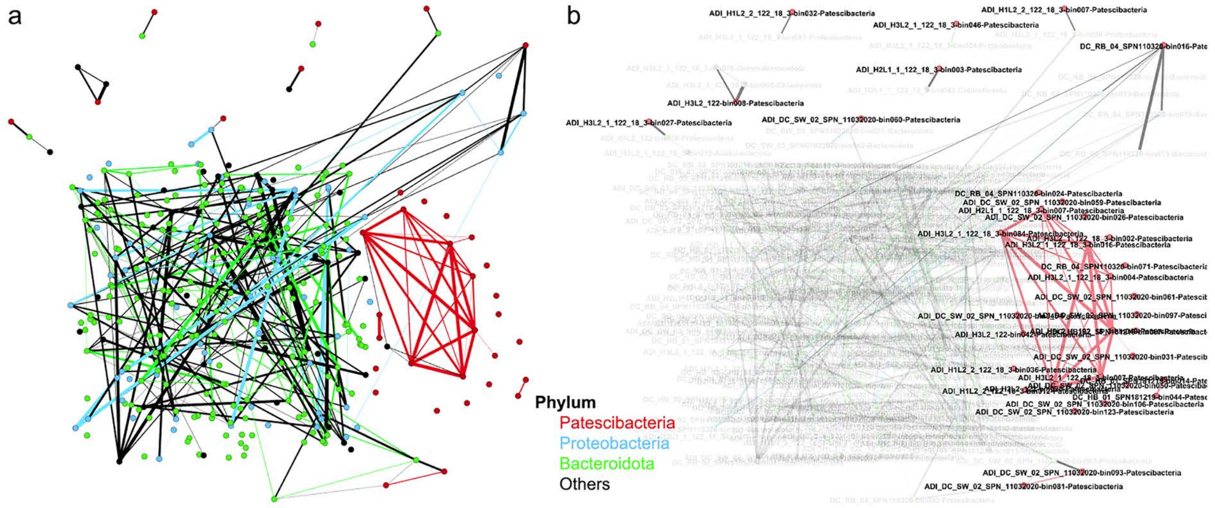

Figure S5: Co-occurrence network of seepage MAGs from this study based on their normalized average genome coverage in seepage metagenomes. **a**, all edges and nodes are visible. **b**, only edges directly connecting to CPR MAGs are visible and CPR nodes are labeled.

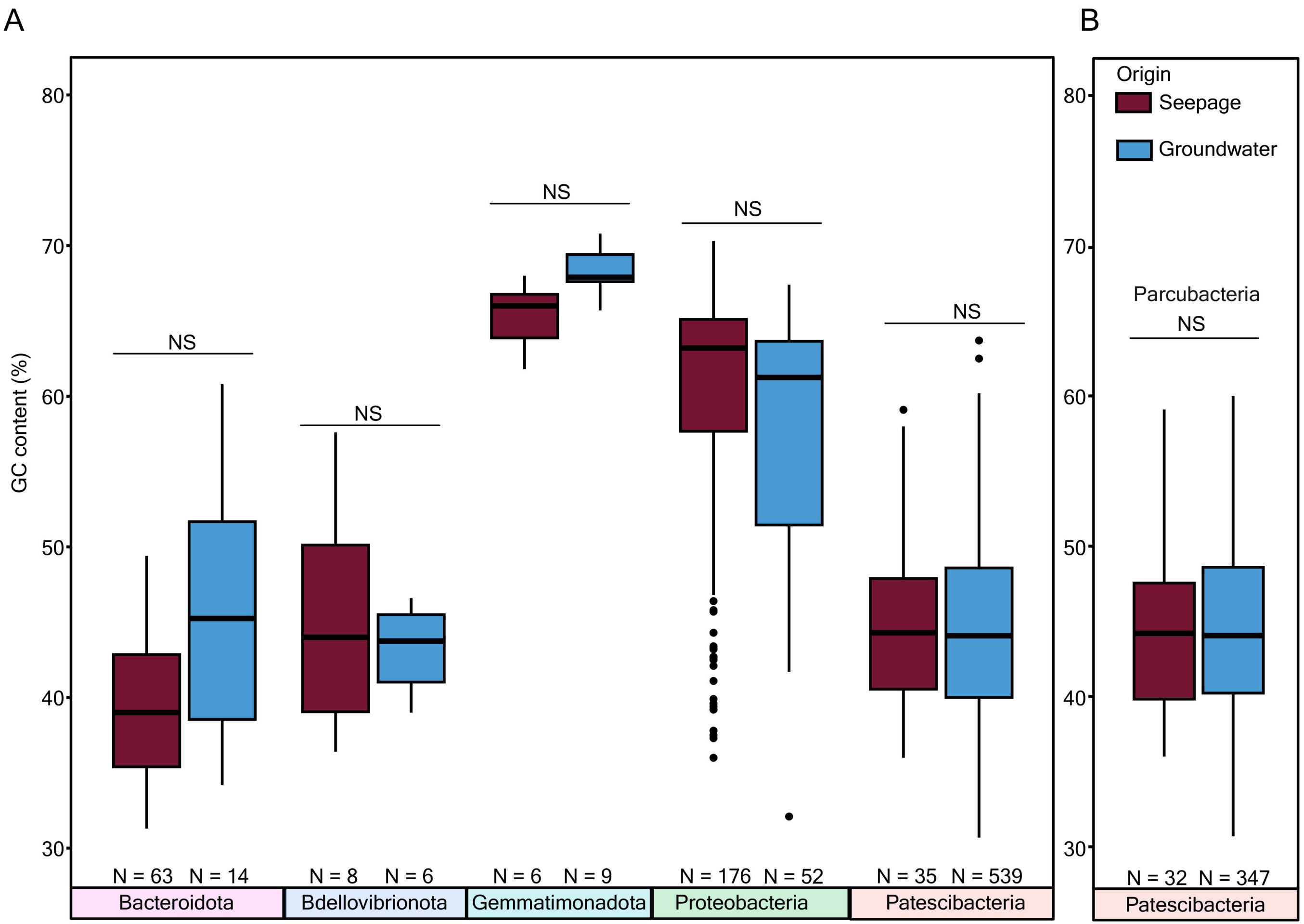

Figure S6: Comparison of genomic GC contents of MAGs generated from seepage and groundwater samples for bacterial phyla (A) and CPR Parcubacteria class (B).
